# Chloropicrin Emission Reduction by Soil Amendment with Biochar

**DOI:** 10.1371/journal.pone.0129448

**Published:** 2015-06-15

**Authors:** Qiuxia Wang, Dongdong Yan, Pengfei Liu, Liangang Mao, Dong Wang, Wensheng Fang, Yuan Li, Canbin Ouyang, Meixia Guo, Aocheng Cao

**Affiliations:** 1 Plant Protection Institute of Chinese Academy of Agricultural Sciences, State key Laboratory for Biology of Plant Disease and Insect Pests, Beijing, China; 2 U.S. Department of Agriculture—Agricultural Research Service (USDA-ARS), San Joaquin Valley Agricultural Sciences Center, Water Management Research Unit, Parlier, California, United States of America; Sun Yat-Sen University, CHINA

## Abstract

Biochar has sorption capacity, and can be used to enhance the sequestration of volatile organic contaminants such as pesticides in soil. Chloropicrin (CP) is an important soil fumigant for the production of many fruit and vegetable crops, but its emissions must be minimized to reduce exposure risks and air pollution. The objective of this study was to determine the capacity of biochar to adsorb CP and the effect of biochar amendments to soil on CP emission, concentration in the soil gas phase, degradation in soil and CP bioactivity for controlling soil borne pests. CP emission and concentration in the soil air phase were measured from packed soil columns after fumigant injection at 20-cm depth and application of selected doses of biocharto the surface 5 cm soil. Laboratory incubation and fumigation experiments were conducted to determine the capacity of biochar to adsorb CP, the effects on CP degradation and, separately, CP’s bioactivity on soil borne pests in soil amended with biochar. Biochar amendment at 2% to 5% (w/w) greatly reduced total CP emission losses by 85.7% - 97.7% compared to fumigation without biochar. CP concentrations in the soil gas-phase, especially in the top 5 cm of soil, were reduced within 48 h following application. The half-life of CP decreased from 13.6 h to 6.4 h as the biochar rate increased from 0% to 5%. CP and its metabolite (dichloronitromethane) both degraded more rapidly in pure biochar than in soil. The biochar used in the present study had a maximum adsorption capacity for CP of less than 5 mg g^-1^. There were no negative effects on pathogen and nematode control when the biochar used in this study was less than 1% (on a weight basis) in soil. Biochar amendment to soil reduced the emissions of CP. CP concentrations in the top 5 cm of soil gas-phase were reduced. CP degradation was accelerated with the addition of biochar. The biochar used in the present study had a low adsorption capacity for CP. There were no negative effects on pathogen and nematode control when the biochar amendment rate was less than 1% (by weight). The findings would be useful for establishing guidelines for biochar use in soil fumigation.

## Introduction

Biochar is a carbon-enriched porous material that is distinguished from other charcoals by its intended use as a soil amendment. Biochar is produced by heating organic material under conditions of limited or no oxygen [1]. Several studies have shown that biochar in soils can enhance the sequestration of organic contaminants. For example, biochar amendment is able to immobilize atrazine in contaminated soils [2]. Moreover, biochar can be applied as a soil improving agent to reduce the potential environmental risks to aquatic environments from simazine [3], glyphosate [4], (4-chloro-2-methylphenoxy)acetic acid (MCPA) [5], (2,4-dichlorophenoxy)acetic acid (2,4-D) and acetochlor [6]. Several studies have shown that biochar produced at low temperature, if applied properly in soil, may be useful for extending the efficacy of pesticides while controlling their potential pollution [6, 7].

Chloropicrin possesses the property of rapid diffusion through agricultural soils and selective toxicity to soilborne pathogens, weeds and pests [8–10]. For its broad biocidal properties, chloropicrin is used today as a pre-plant soil fumigant primarily in high-valued horticultural crops such as strawberries [11], peppers [12] and vegetables [13, 14]. The US Environmental Protection Agency (EPA) completed a comprehensive and exhaustive eight-year review of chloropicrin and concluded that the chemical could continue to be used safely by farmers in US agriculture [15]. CP has also been identified as an acceptable alternative to methyl bromide which is being phased out as a soil fumigant due to its effect on stratospheric ozone [16]. However, CP is susceptible to rapid emission losses after being applied to soil due to its high volatility [17]. Like other fumigants, high levels of CP emissions may endanger the health of workers and bystanders and also contribute to volatile organic compounds in the air that adversely affect air quality [18]. Chloropicrin is a powerful eye irritant and its effect diminishes with time after exposure. Dangers arise from inhalation of chloropicrin, and extended exposure can lead to difficulty in breathing [19]. It has been reported that chloropicrin can induce endoplasmic reticulum stress in human retinal pigment epithelial cells [20].

Recent studies have shown that CP emissions may be reduced through the use of plastic barrier films laid on the soil surface [18, 21], organic amendments [22], deep injection [17], ammonium thiosulfate amendments [17], and water treatments on the soil surface [22]. Although much experimental information has been obtained, the results in emission reduction varied significantly and research gaps remain [23].

A study showed that amendment of surface soil with 0.5% or more biochar reduced total 1,3-dichloropropene (1,3-D) emission loss by > 92% compared with fumigation without biochar, and the reduction in 1,3-D emissions was associated with the adsorption properties of biochar [24]. The amendment of surface soil with biochar shows a potential for reducing fumigant emissions while at the same time benefiting agricultural production. The objective of this study was to determine the amount of CP adsorbed on biochar and the effect of biochar applied to the soil surface on CP emission reduction, the potential impact on CP concentration in the soil air phase, CP degradation in soil and CP bioactivity for soil borne pests.

## Materials and Methods

### Soil, biochar and chemicals

With the authorization of the institute of Haidian agricultural science, Beijing, soil samples used in the laboratory studies were collected from the top 20 cm of a greenhouse soil in Tujing village, Xibeiwang town, Haidian district, northwest of Beijing. The greenhouse site did not involve endangered or protected species and the GPS coordinates were 40°03’33.2”N, 116°15’46.2”E. And there were no specific permissions required for the greenhouse site. The soil was a sandy loam (57.9% sand, 38.9% silt, and 3.2% clay) with an organic carbon content of 2.8% and pH 7.8. The soil was air dried and sieved through a 2 mm screen before use in the laboratory studies.

Biochar used in the study was obtained from Lijiachang Biogas Station (Beijing, China). The biochar was derived from miscellaneous pieces of wood pyrolyzed at 400°C for 4 h. The biochar was processed with a universal high-speed smashing machine (FW80, Tianjin Taisite Instrument Co., Ltd., Tianjin, China) in the laboratory before use. The specific surface area (SSA) of the biochar was measured using a porosimetry analyzer (Model V-Sorp 2800P, Gold APP Instruments Corp., Beijing, China) and found to be 7.69 m^2^ g^-1^. Other physical/chemical characteristics of the biochar used in this study are listed in Table 1.

**Table 1:** Physical/chemical characteristics of biochar used in this study.

C (%)	N (%)	P (%)	K (%)	O (%)	H (%)	pH	CEC^a^ (cmol·kg^-1^)	SSA (m^2^ g^-1^)
62.33	2.00	0.43	1.43	5.41	1.85	9.23	12.55	7.69

^a^CEC: cation exchange capacity.

Analytical grade chloropicrin (99.5%) was obtained from Dow AgroSciences (Indianapolis, IN, USA). Commercial chloropicrin (99.5%) was purchased from Dalian Lvfeng Chemical Co. Ltd (Dalian, China). Analytical grade hexane, ethyl acetate and sodium sulfate anhydrous (NaSO_4_) were obtained from Beijing Chemical Works (Beijing, China). Polyethylene film (PE, thickness, 0.06 mm) was supplied by Baoding Baoshuo Plastic Co. Ltd (Hebei Province, China).

### Soil column experiment

**Fig 1:**
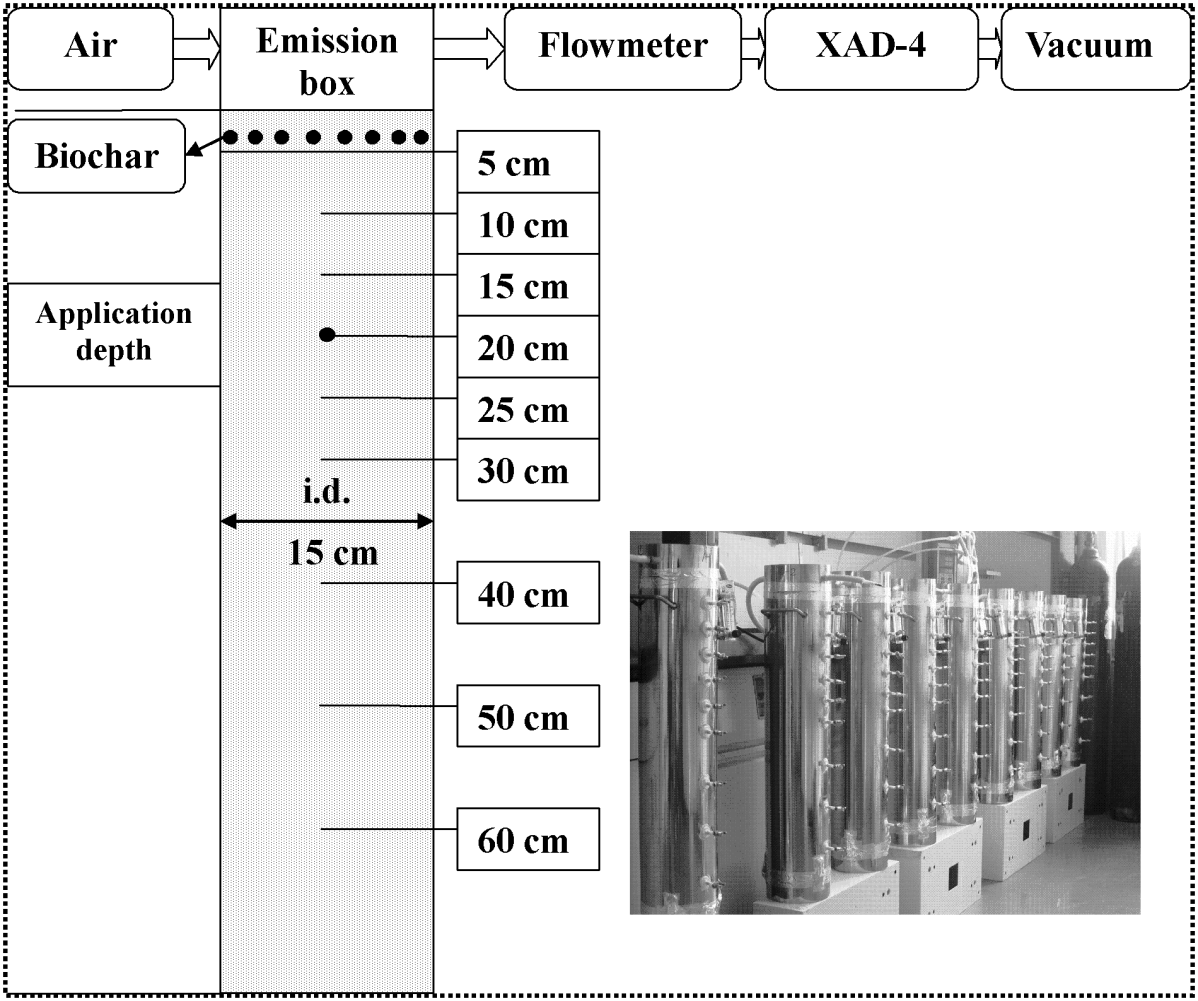
Schematic diagram of the soil column.

Stainless steel soil columns (15 cm diameter × 72 cm length) were used in the study. The design and setup of the columns were similar to the description in Zhang and Wang [25]. A schematic plot of the soil column is shown in Fig 1. The column bases were sealed with steel sheets, with nine sampling ports located at 5, 10, 15, 20, 25, 30, 40, 50, and 60 cm soil depths. The top of each column was sealed with a stainless steel flow through flux chamber (6.5 cm height). An air flow rate of 100 ± 10 mL min^-1^ was applied to the flux chamber to sweep volatilized CP through XAD sorbent tubes (Amberlite XAD-4; Supelco, Bellefonte, PA). Soil (water content 12.5% w/w) was packed into the columns to a bulk density of 1.3 g cm^-3^. Pre-weighed (0, 23.0 and 57.4 g) biochar was uniformly mixed into the top 5 cm layer of the soil column before packing. This corresponded to biochar rates of 0%, 2% and 5% (w/w) in the top 5 cm soil. After soil was packed into the columns, PE tarp was installed in each column between the soil surface and the chamber to simulate PE tarp agricultural practices. The PE film was first sealed to the edges of the stainless steel columns using sealant-coated aluminum tape to minimize leakage. The column and the chamber were then sealed together using aluminum foil tape. Two mL CP-methanol solution (268 g L^-1^) was injected in the column through the sampling port located at 20 cm soil depth. Each treatment was repeated three times. Monitoring and sampling of experiments lasted for 623 hours. The experimental temperature ranged from 25 to 32°C.

After 1.5, 6, 12, 24, 48, 72, 96, 120, 144, 168, 192, 216, 264, 312 and 408 h, soil gas was sampled and determined for CP. A 0.5 mL sample of soil gas was collected from the sampling ports using a gastight syringe. The gas sample was dispensed into a 20 mL clear headspace vial which was immediately crimp-sealed with an aluminum cap and Teflon-faced butyl-rubber septum (Agilent Technologies, Palo Alto, CA, USA). This method has been confirmed to be accurate and reproducible [26]. 0.2 g of sodium sulfate was added to each vial before sample injection in order to minimize any moisture effect on fumigant stability. The headspaces of the vials containing soil gas samples were analyzed using an Agilent 7890A gas chromatograph (GC) coupled with an Agilent 7694E headspace sampler and a micro electron capture detector (Agilent Technologies Inc., Palo Alto, CA, USA). An HP-5 capillary column (30 m length × 0.25 mm i.d. ×0.25 μm film thickness, Agilent Technologies) was used for analysis. The detector and inlet temperatures were 250 and 150°C, respectively. The oven temperature was held at 90°C for 8 min. The autosampler headspace conditions were as follows: 1.0-mL sample loop; 90, 93, and 95°C for sample equilibration, loop, and transfer line temperatures, respectively; 5-min vial equilibration time; 0.5-min loop filling time; 0.05-min loop equilibration time; 0.1-min pressurization time; 0.5-min injection time; 56.6-kPa vial pressure; and low shake mode for 1 min. The split ratio was 150/1.

The XAD tubes for absorbing CP evaporating from the flux chamber were replaced every 2–3 h during the first 72 h. Incrementally longer sampling interval times were used in the later part of the experiment. All samples were stored at −80°C before analysis. The XAD sorbent tubes were broken, and all materials were transferred into 20 mL clear headspace vials. Eight milliliters of hexane were added, and the vials were immediately capped with a Teflon-faced septum. Ten sample was extracted with a shaker at 800 g min^-1^ for 1 h and the supernatants were then filtered into a 2 mL vial using a 0.22 μm Nylon syringe filter. For samples collected during 2–72 h, aliquot of the filtered solutions were diluted to 10 times with hexane before being injected into the GC described above, and coupled with an Agilent 7683B automatic liquid sampler. The chromatographic conditions were the same as for the soil gas samples, except that the inlet temperature was 250°C and the injected liquid volume was 1 μL.

Upon termination of the experiment, soil samples for CP residue and soil water content determination were taken from five depth increments: 0–5, 5–15, 15–30, 30–50 and 50–72 cm from each column. CP extraction from soil samples was conducted using the procedure reported in Chellemi et al. [27]. An equivalent dry weight of 8 g of soil was added in a 20 mL clear vial, then extraction with ethyl acetate involved shaking soil with 8 g anhydrous sodium sulfate and 8 ml ethyl acetate on a mechanical shaker (800 g min^-1^ for 1 h). The supernatants were then filtered using a 0.22 μm Nylon syringe filter into a 2 mL chromatography vial and analyzed using the same chromatographic conditions as for XAD extracts. The amount-known CP standards in activated carbon were extracted by the same extraction method. The recovery of CP was over 91%, which means the majority of CP could be extracted from soil or biochar.

The fate or mass balance of the applied CP in soil with or without biochar was estimated from cumulative emissions, amounts in the soil gas phase and residues in soil samples that were taken at the end of experiments.

### CP degradation in biochar amended soils

A laboratory incubation experiment was conducted to determine the effect of biochar amendments on the degradation of CP, using a method similar to that reported in Qin et al. [28]. The soil at 12.5% water content (w/w) was amended with biochar at rates of 0, 2 and 5% (w/w). Eight grams of amended soil were placed in a 20 mL clear headspace vial, 5 μL of ethyl acetate containing 150 g L^-1^ of CP was added to each vial. The initial concentration of CP in soil was 93.75 μg g^-1^. Vials were immediately sealed with an aluminum cover and Teflon-faced septa after fumigation application. The treated vials were then inverted and placed in incubators at 30°C. After incubation for 1, 3, 5, 7, and 10 days, three replicate samples from each treatment were removed from the incubator and immediately stored at -80°C until CP concentration analysis. CP was extracted and analyzed from soil samples using the same methods with soil samples for residue determination.

### Degradation products of CP in soil and biochar

One gram of soil or biochar was placed in a 20 mL clear headspace vial, and 5 μL of ethyl acetate containing 150 g L^-1^ of CP was added to each vial. The treated vials were then inverted and placed in incubators at 30°C for 0.5, 1, 3, 6 and 12 h. Degradation products were extracted from soil samples using the same methods with soil samples for CP residue determination. The extracted degradation products were determined by GC-MS analyses performed on an Agilent 7890A GC System equipped with a split/splitless injection port and a 5975C mass selective detector. The separation was performed on a DB-5MS (30 m×0.25 mm ID×0.25 μm film thickness) column (Agilent J&W Scientific, USA). Helium (99.999%) was used as carrier gas and maintained at a flow rate of 0.8 mL/min. The injection port temperature was 250°C and splitless mode was used until 1 min after injection. The initial oven temperature was 40°C held for 4 min, then programed to 100°C at 15°C /min, followed by a ramp of 30°C /min to 250°C. The total run time was 13.0 min. The quadrupole, ion source and transfer line temperatures were 150°C, 230°C and 280°C, respectively. Electron impact ionization (EI) with energy of 70 eV and an electron multiplier voltage of 1494 V were used. The full scan mode with scan range of m/z 50–250 and a solvent delay of 3.6 min were used to obtain the MS spectrum. MSD Productivity ChemStation software (Version E.02.00.493) was used to control instrument and acquire data.

### Adsorption experiment

**Fig 2:**
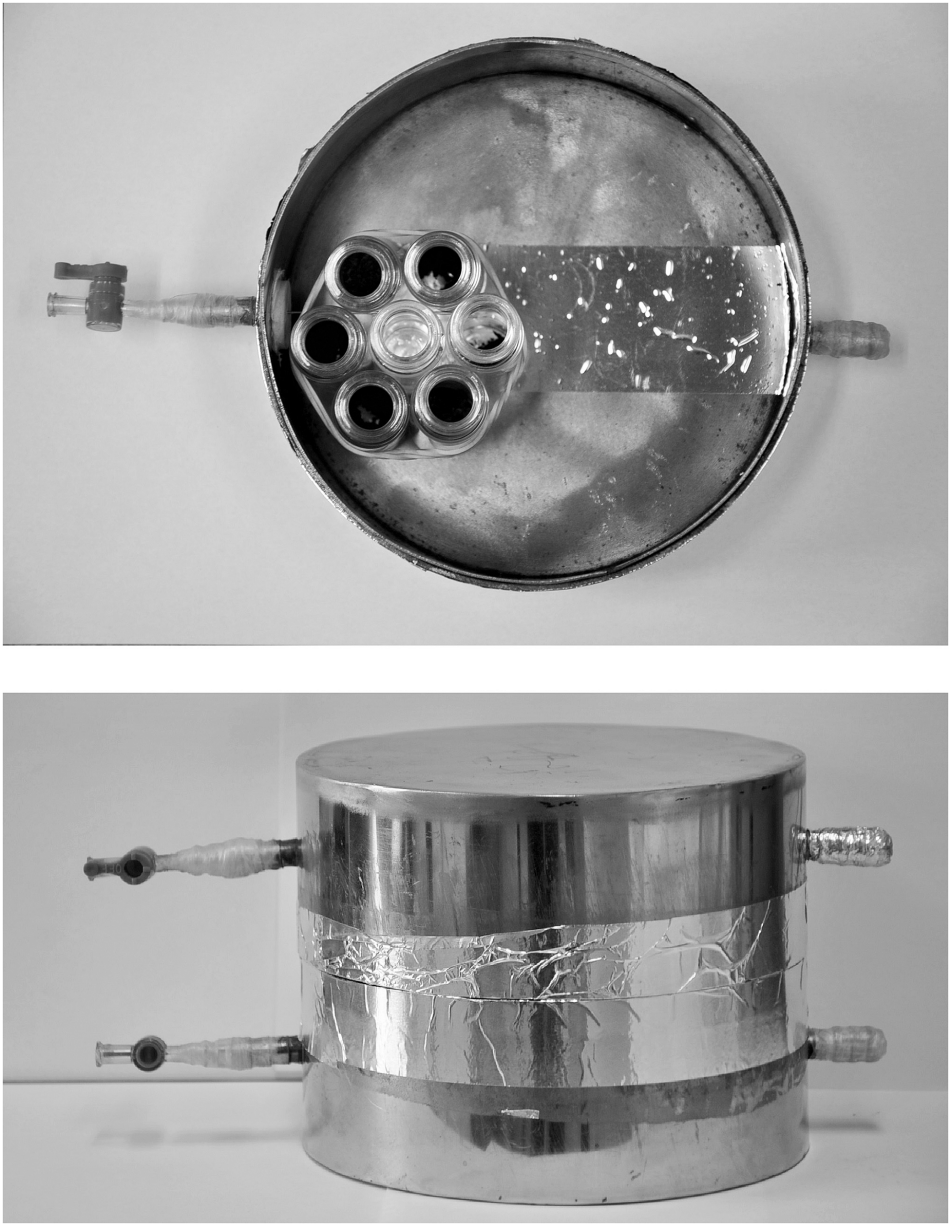
Adsorption configuration.

0.15 g biochar or activated carbon was weighed into a 20 ml clear headspace vial. Three replicates (total of 6 vials for each time) were put into a container. The container was constructed of two stainless steel cylindrical boxes, each 15 cm ID and 4 cm high (Fig 2). Five mL of pure CP liquid were injected to a 20 ml clear headspace vial and put into the container to generate a saturated vapor phase. The container was sealed by aluminum tape after the vials were put into the container. Then the containers were placed in incubators with temperatures settled at 30°C. After incubation for 3, 6, 9, 12 and 24 hours, the containers were removed from the incubator. The container was opened and ventilated for about 30 minutes in a fume hood. Then the biochar or activated carbon was analyzed using the same method as already described.

### CP efficacy in biochar amended soils

Soil was collected from the top 20 cm of a commercial greenhouse at Fangshan district, Beijing, China (39°32’33.5”N, 115°48’26.1”E) which was heavily infested with the root-knot nematodes and pathogens. The field site did not involve endangered or protected species and the GPS coordinates were 39°32’33.5”N, 115°48’26.1”E. The soil was a sandy loam (83.4% sand, 15.1% silt, and 1.6% clay) with an organic matter content of 1.5% and pH 7.6. The soil was amended with biochar at rates of 0, 0.5, 1, and 2% (w/w). After thorough mixing with the biochar amendments, 300 g samples were placed in 500 mL glass jars. CP liquid of 1.8, 3.6, and 7.2 μL was added to jars to generate CP rates in soil of 10, 20 and 40 mg kg^-1^ soil. Jars were immediately sealed with an airtight lid after CP application and placed in a constant temperature room at 30°C. Untreated control (CK, jars containing the soil without biochar and CP) was placed in the same conditions with the treated jars. Experiments were conducted with five replicates. After 4 days fumigation, the nematodes and fungal pathogens in the soils were separated for evaluating CP’s fumigation efficacy in biochar amended soils. Root-knot nematodes were separated from 100 g soil subsamples by centrifugation. The semiselective solid medium described by Masago et al. [29] was used for *Phytophthora* detection: selective inhibition of *Pythium* spp. on medium for direct isolation of *Phytophthora* spp. from soils. A semiselective medium described by Komada was used for *Fusarium* spp. detection: development of selective medium for quantitative isolation of *Fusarium* spp. from natural soil [30].

### Data analysis

The degradation of CP in the soil amended with biochar at rates of 0, 2 and 5% (w/w) was described using first-order kinetics regression:

$$C_{t}=C_{0}e^{\text{-}k/t}$$ (1)

Where *C*_*0*_ is initial concentration of CP in soil (93.75 μg g^-1^) and *C*_*t*_ is CP concentrations (μg g^-1^) in soil at time *t* (h) after CP application; *k* is the first order rate constant (h^-1^).

On the basis of Eq (1), the half-life of the fumigant in soil or biochar was calculated as follows:

$$t_{1/2}=\frac{0.693}{k}$$ (2)

The observed efficacy (*E*_*0*_) on pathogens after fumigation was calculated as

$$E_{\text{0}}=(1-\frac{P_{T}}{P_{CK}})\times 100$$ (3)

Where *P*_*CK*_ is the population density of a pathogen in the untreated control and *P*_*T*_ is the population density of a pathogen in the treated soil.

The efficacy (*E*) on root-knot nematodes after fumigation is calculated as

$$E=(1-\frac{{\text{S}}_{T}}{{\text{S}}_{CK}})\times 100$$ (4)

Where *S*_*CK*_ is the number of surviving root-knot nematodes in the untreated control and *S*_*T*_ is the number of surviving nematodes in the treated soil.

Pest control efficacy data were analyzed statistically according to Duncan’s Multiple Range Test using Statistical Analysis System (SAS) computer software.

## Results and Discussion

### Emission flux

**Fig 3:**
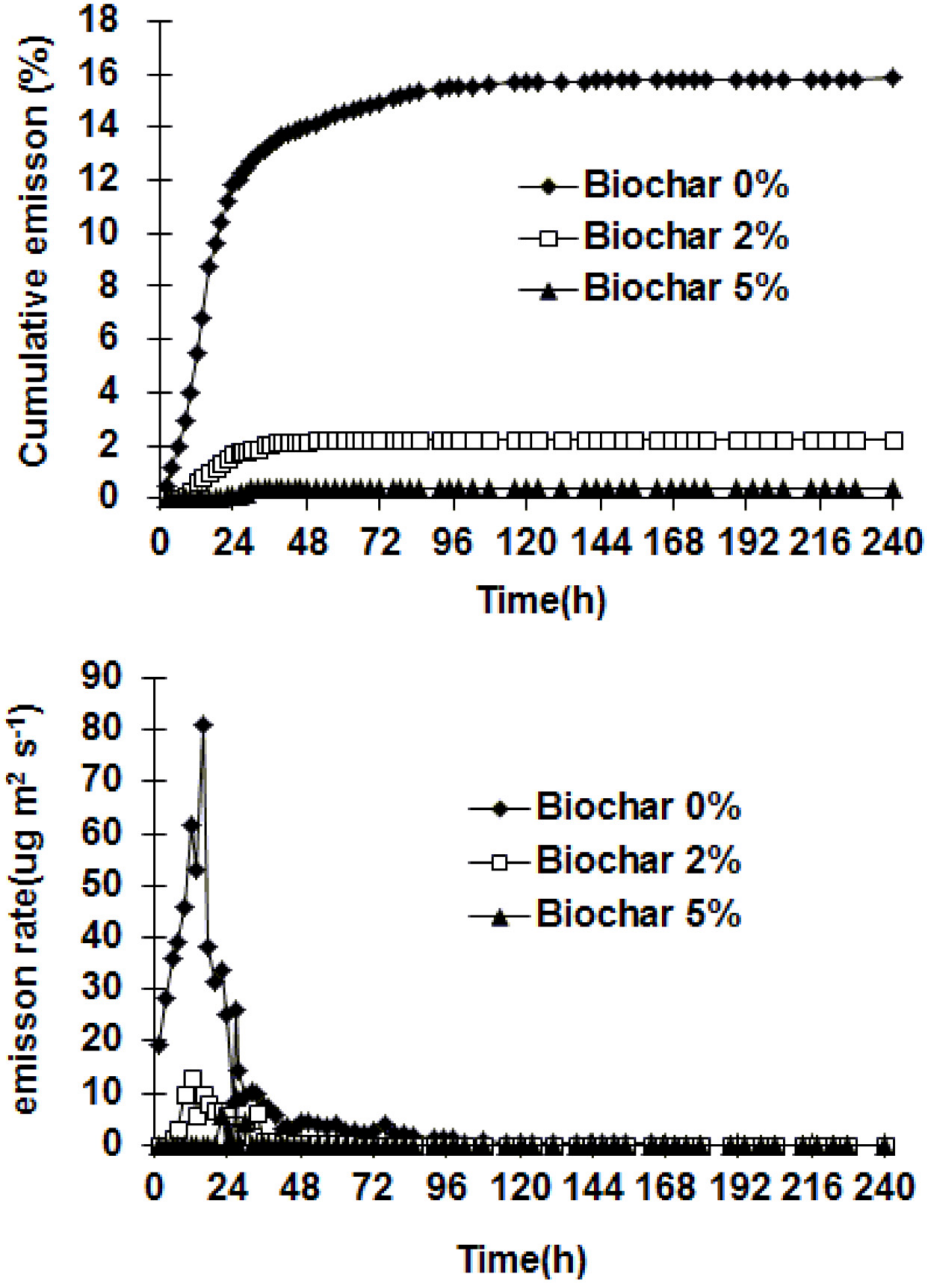
Emission rates and cumulative emissions of CP in soil column treatments.

The emission fluxes of CP from the column treatments are shown in Fig 3. In the biochar 0% treatment, the emission flux was 19.3 μg m^-2^ s^-1^ at the first determination time, i.e. 2 h after CP application. The emission flux initially increased with time, exhibiting the peak flux (80.9 μg m^-2^ s^-1^) at 16 h after injection. The flux subsequently declined with time, and reached the lowest value (0.02 μg m^-2^ s^-1^) at 240 h after application. In the biochar 2% treatment, the initial emission flux was 0.01 μg m^-2^ s^-1^, increasing with time to a maximum emission flux of 9.9 μg m^-2^ s^-1^ at 16 h after injection. The flux then declined with time, falling to 0.001 μg m^-2^ s^-1^ at 240 h after injection. In the biochar 5% treatment, at 22, 27 and 30 h after CP application, the emission fluxes were 5.6, 9.5 and 4.4 μg m^-2^ s^-1^, respectively. At other determination times, the fluxes were all lower than 0.01 μg m^-2^ s^-1^.

### Cumulative emissions

Cumulative emission losses of CP from the column treatments are shown in Fig 3. The emission percentages (percent of total CP applied) over the entire study were 15.9% for biochar 0% treatment, 2.3% for biochar 2% treatment and 0.4% for biochar 5% treatment. The above results indicated that biochar amendment at a percentage of 2% to 5% was able to reduce total fumigant emission losses by 85.7%- 97.7% compared with fumigation without biochar. The results demonstrated that the biochar amendment in surface soil reduced CP emission losses effectively.

### CP concentration in soil-gas phase

**Fig 4:**
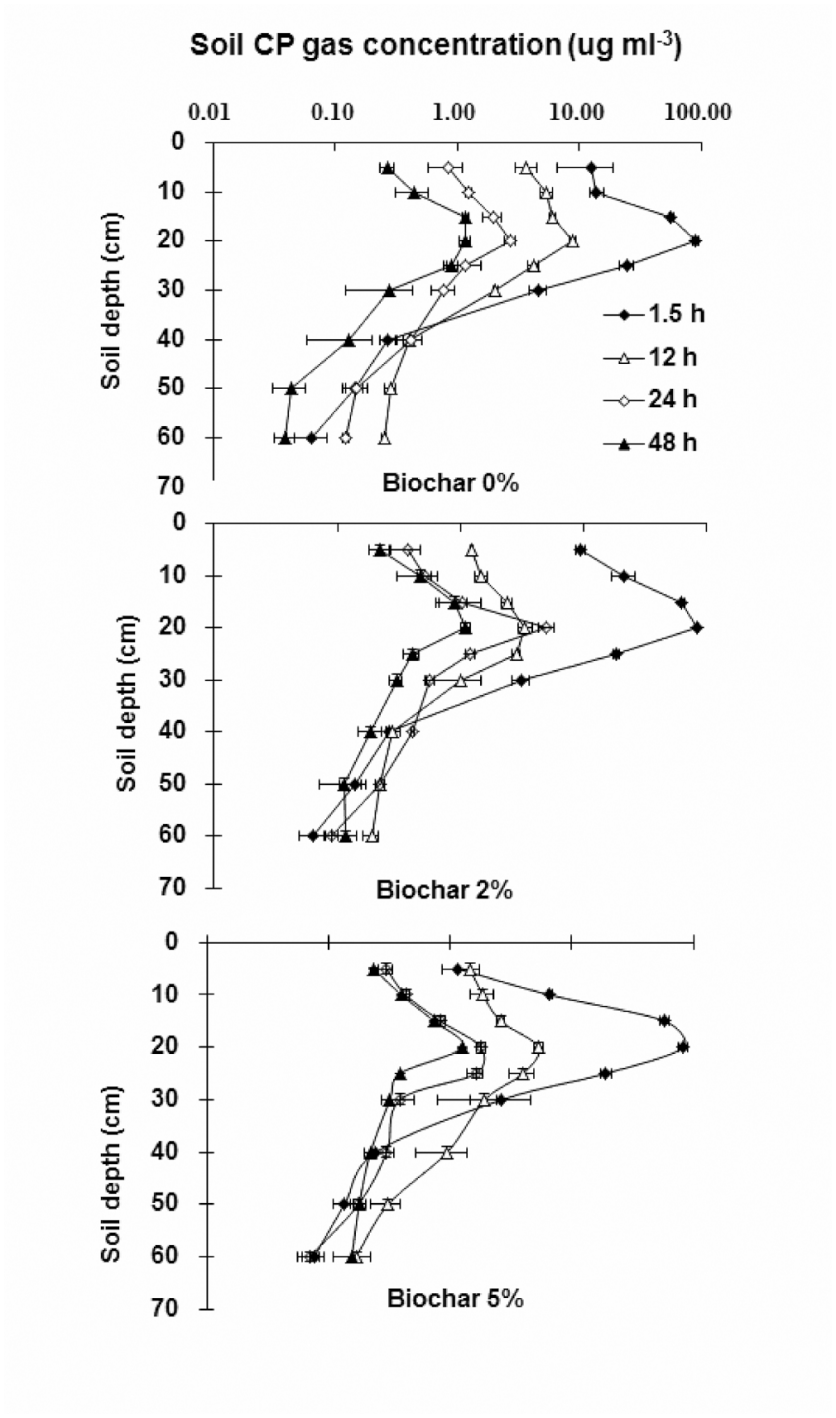
Concentration of CP in the soil-gas phase in soil column treatments.

The distributions of CP in the soil-gas phase at 1.5, 12, 24 and 48 h after application are shown in Fig 4. At 1.5 h after CP injection, the concentrations at 5 cm depth were 12.8, 9.5 and 1.2 μg mL^-3^, and at 10 cm depth were 14.0, 21.4 and 6.7 μg mL^-3^ for biochar 0, 2 and 5% treatments, respectively. There were no significant differences between concentrations at other soil depths for all three treatments. At 12 and 24 h after application, the concentrations above the 10-cm depth for 2 and 5% biochar treatments were half that of biochar 0% treatments. At 48 h after injection, however, differences in CP concentration in the soil-gas phase were not significant among the three treatments.

The results indicated that biochar adsorption or degradation may have been responsible for the reduction in CP concentration. Adsorption or degradation requires a certain time to reach equilibrium, and the CP concentration or total amount would not be affected after equilibrium. The reason for the concentration reduction needed to be verified by a degradation and adsorption experiment, and the results are given in the next section.

### CP residue and losses in soil

**Fig 5:**
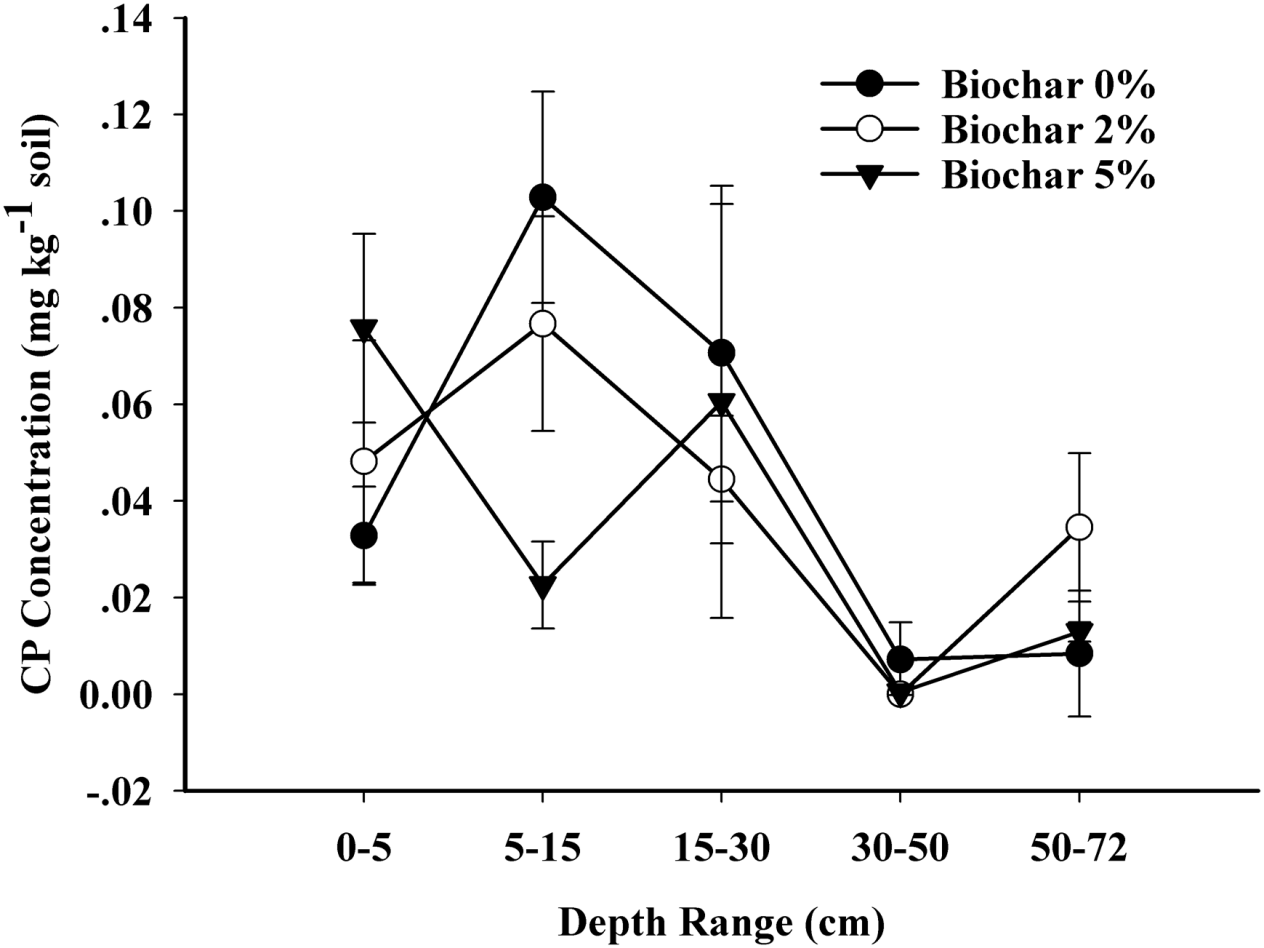
Residue of CP in soil in soil column treatments.

Residual CP in the soil (solid) and liquid phases was measured at the end of the experiment (Fig 5). Although residual CP was low in all soils, higher concentrations were observed at 0–5 cm depth in soil with biochar amendment, likely because of increased binding to biochar. The CP residue was lower in soil amended with biochar than in pure soil at 5–15 cm depth, indicating that CP tended to move into soil with biochar amendment and possibly bound with biochar.

The estimated percentages of CP losses (Table 2) were 83.85%, 97.44%, and 99.30% for the biochar treatments of at 0, 2, and 5% level, respectively. The results indicated that higher biochar amendment led to greater CP degradation in soil.

**Table 2:** Fate of CP after application to soil columns.

Treatments	Emission^a^	Soil/liquid phase^a^	Gas phase^a^	Losses^b^
Biochar 0%	15.9	0.10	0.15	83.85
Biochar 2%	2.3	0.09	0.17	97.44
Biochar 5%	0.4	0.07	0.23	99.30

^a^ The measured CP amount as a percentage of the applied amount.

^b^ Calculated by deducting the measured CP from the applied amount.

### Dynamic degradation of CP in biochar amended soil

The degradation rate constant (*k*) and half-life (t_1/2_) are listed in Table 3. In biochar amended soil, the first-order kinetics model simulated the degradation of CP very well; the correlation coefficient values were all higher than 0.9999. The half-life decreased from 13.6 h to 6.4 h and the first-order rate constant significantly increased from 0.05 to 0.1 h^-1^ as the biochar amendment rates increased from 0% to 5%. The results showed that biochar amendments decreased the half-life of CP in soil, and the degradation of CP would accelerate when increasing the amount of biochar amendment.

**Table 3:** Effects of biochar on CP degradation in soil^a^.

Treatments	Degradation rate constant *k* (h^-1^)	Half-life t_1/2_ (h)	Correlation coefficient r^2^
Biochar 0%	0.051 ± 0.008 c^b^	13.6	0.9999
Biochar 2%	0.079 ± 0.002 b	8.8	1.0000
Biochar 5%	0.108 ± 0.011 a	6.4	1.0000

^a^Using first-order kinetics regression.

^b^Values are means ± standard errors (n = 3). Different letters indicate statistical significance at *P* = 0.05 level using the Duncan’s multiple range test.

### Degradation products of CP in soil and biochar

Chloropicrin was detected in soil and biochar at retention time of 5.32 min. Its metabolite dichloronitromethane was also found at retention time of 4.78 min, and confirmed by comparing its MS spectrum in samples to that in NIST liberary. Cole et al. [31] has investigated the degradation mechanisms of CP. Their results suggest that the hydroxyl radical adds to the nitro-group, while the hydrated electron reacts via dissociative electron attachment to give two carbon centered radicals. Based on the study of Cole et al. [31], the processes listed below would be primarily responsible for the destruction of CP:

$$CP\;+\;e_{aq}{}^{-}\to^{•}CCl_{2}NO_{2}+\;Cl^{-}$$

$$CP\;+\;e_{aq}{}^{-}\to^{•}CCl_{3}+\;NO_{2}{}^{-}$$

$$CP\;{+}^{•}OH\;\to \;intermediate\;\to^{•}CCl_{3}+\;ONOOH$$

$$CP\;+\;H^{•}\to^{•}CCl_{2}NO_{2}+\;H^{+}+\;Cl^{-}$$

$$CP\;+\;H^{•}\to^{•}CCl_{3}+\;H^{+}+\;Cl^{-}$$

$$CP\;+\;O_{2}{}^{-•}\to^{•}CCl_{2}NO_{2}+\;Cl^{-}+\;O_{2}$$

**Fig 6:**
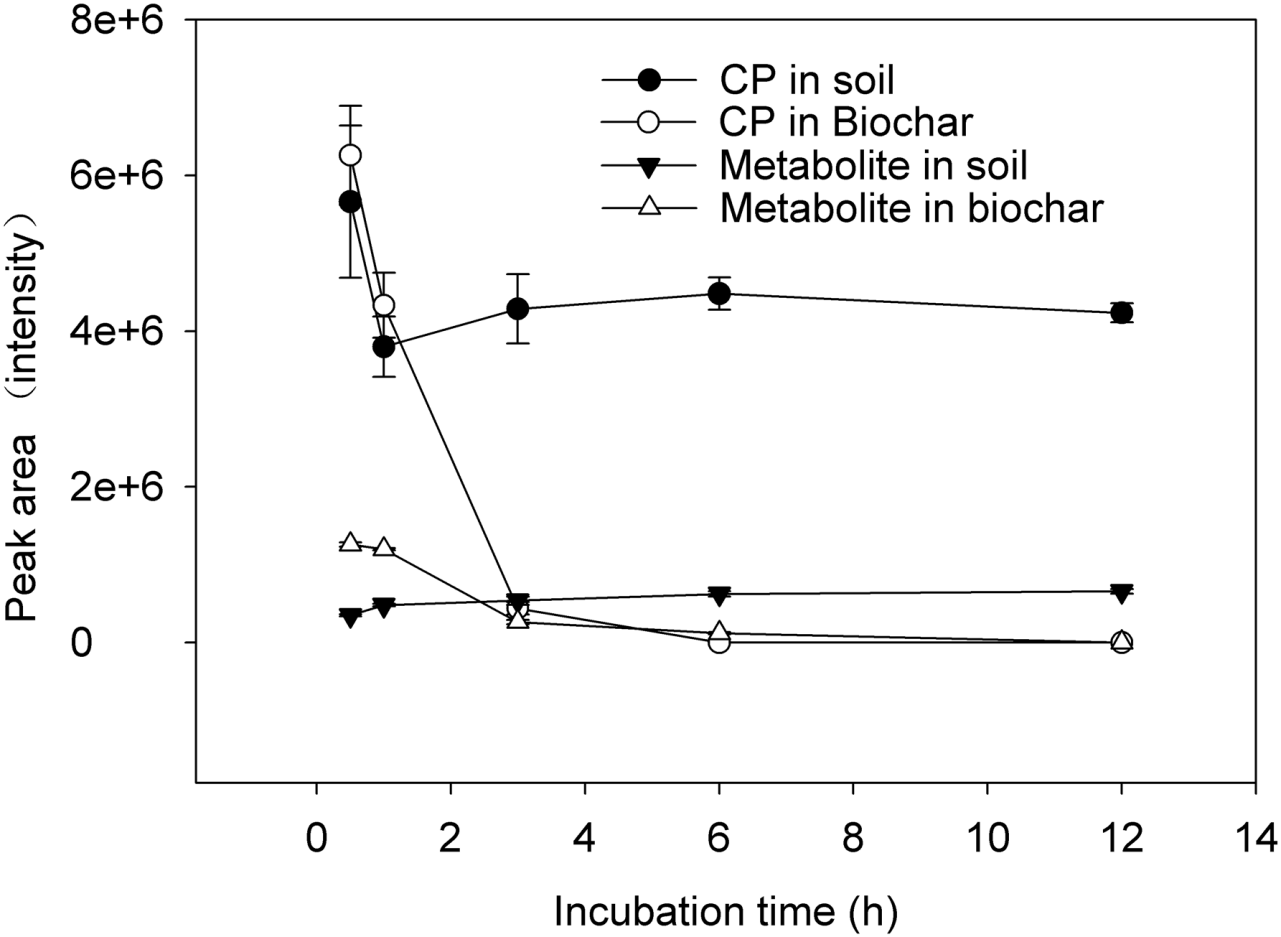
Changes in concentration of CP and dichloronitromethane in soil and in biochar.

The above process shows that dichloronitromethane is a major metabolite of chloropicrin. In the present study, the peak areas (Fig 6) of extracted ion of CP and its metabolite were used as an indicator of concentration change in sample extracts. The concentrations of CP in soil and in biochar were similar after incubation for 0.5 h, however the concentration of metabolite in biochar was 2.6 times higher than in soil. The concentration of CP and its metabolite degraded rapidly in biochar with increasing time, and they could not be detected after incubation for 6 h and 12 h, respectively. CP concentration decreased rapidly until 1 h following application in soil, and then there was no obvious change in concentration between 1 h to 12 h after incubation. The concentration of dichloronitromethane increased in soil with increasing time. The above results indicated that CP degraded faster in biochar than in soil.

Biochar contains numerous functional surface groups, such as hydroxyl-OH, keton-OR, ester-(C = O), aldehyde-(C = O)H, nitro-NO_2_, and carboxyl-(C = O)OH groups [32, 33]. Surface oxidation led to a high charge density on surfaces of biochar particles [34]. Using electron spin resonance Meszaros et al. [33] observed that an abundance of organic radicals exist in biochar, and the quantity of radicals increases with decreasing production temperature. CP degradation would be accelerated by reaction with functional surface groups of biochar. CP could react with biochar in surface soil when the chemical diffuses from the deeper soil to the surface soil, and this may be one of the reasons that biochar amendments reduced the CP emission from soil to air.

### Adsorption capacity of biochar to CP

**Fig 7:**
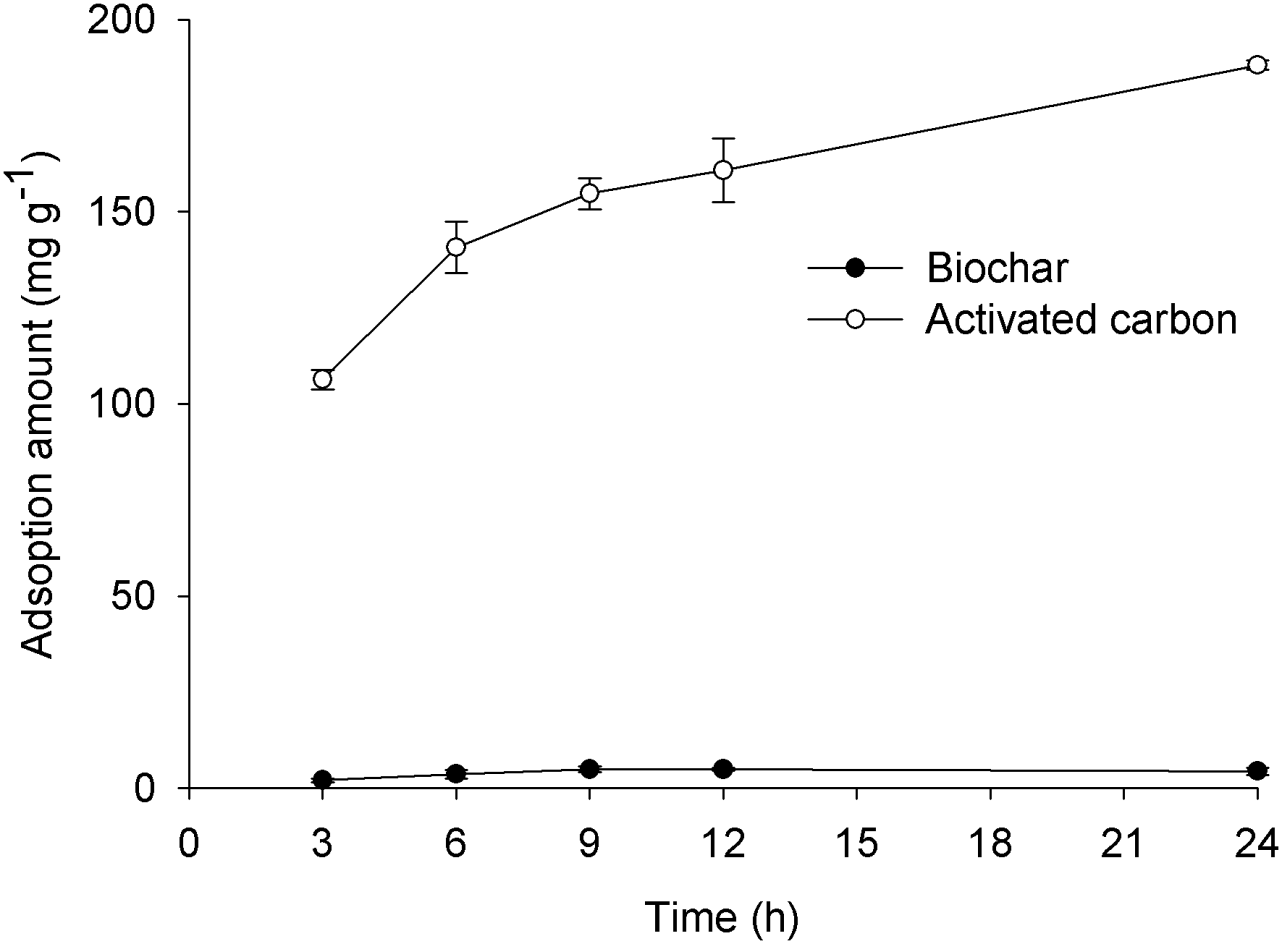
Adsorbed amounts of CP on biochar and activated charcoal.

The amount of CP adsorbed on biochar or activated charcoal increased with increasing time (Fig 7). The amounts adsorbed of CP onto biochar did not change significantly after 9 h incubation. The maximum adsorption capacity of the biochar used in the present study was less than 5 mg CP g^-1^. In contrast, the adsorbed amount of CP adsorbed on activated charcoal was 188 mg g^-1^ at 24 h after incubation, which was significantly higher than biochar. Adsorption and degradation must occur simultaneously when CP meets biochar. The amount of CP kinetic adsorption onto biochar was a balance between adsorption and degradation. The amounts of CP adsorbed onto biochar maybe reached a stable maximum after the functional surface groups of biochar or unknown components reacted totally with CP, which was kept in a saturated condition in the experiment.

### CP efficacy in biochar amended soils

Table 4 shows the efficacy of CP fumigation on *Fusarium* spp., *Phytophthora* spp. and root-knot nematodes in biochar amended soil. Efficacy against *Fusarium* spp. and *Phytophthora* spp. did not differ significantly between the biochar rate of 0, 0.5 and 1%, regardless the CP rates. When the biochar rate was increased to 2%, the efficacies against the two pathogens decreased dramatically to less than 30%. The results indicated that adequate pathogen control was obtained when biochar amendment in soil was up to 1%, however, biochar amendment at rates above 2% may have caused the observed reductions in CP efficacy for *Fusarium* spp. and *Phytophthora* spp. Biochar amendment doses at higher rates (2%) negatively affected nematode control when the CP rate was less than 20 mg kg^-1^, but nematode control was maintained when the CP rate was increased to 40 mg kg^-1^ (Table 4).

**Table 4:** Efficacy of CP fumigation treatments on *Fusarium* spp., *Phytophthora* spp. and Root-knot nematodes in biochar amended soil.

CP rates (mg kg^-1^ soil)	Biochar amendment rates (%, w/w)	*Fusarium* spp. (%)	*Phytophthora* spp. (%)	Root-knot Nematodes(%)
10	0.0	81.48±11.78 a^a^	75.63±15.52 a	88.64±7.09 a
	0.5	86.42±6.53 a	77.06±6.3 a	87.88±9.04 a
	1.0	80.66±7.37 a	72.76±15.59 a	64.39±7.85 b
	2.0	2.06±3.56 b	0.00 b	74.24±9.04 b
20	0.0	97.94±2.57 a	98.21±1.64 a	79.48±6.06 a
	0.5	95.47±4.99 a	92.11±1.64 a	84.85±5.36 a
	1.0	88.45±7.04 a	91.43±2.48 a	85.61±9.04 a
	2.0	6.17±10.69 b	3.23±5.59 b	69.38±7.02 b
40	0.0	98.77±1.23 a	94.98±6.83 a	93.18±7.29 a
	0.5	93.42±5.38 a	88.17±6.72 a	81.82±10.51a
	1.0	91.98±0.87 a	93.55±3.04 a	87.88±15.01a
	2.0	28.67±7.77 b	56.45±3.8 b	82.58±9.88 a

^a^ Values are means ± standard errors (n = 5). Different letters indicate statistical significance at *P* = 0.05 level using the Duncan’s multiple range test.

In summary, biochar amendment to soil reduced the emissions of CP. CP degradation was accelerated with the addition of biochar by possibly reacting with functional surface groups of biochar. CP concentrations in the soil gas-phase were reduced, especially in the top 5 cm of soil, and the present study showed that there were no negative effects on pathogen and nematode control when the biochar amendment rate was less than 1% (by weight). So as a fertilizer or as a method to reduce emission of CP, the biochar amendment rate of 1% is recommended for use in fields which will be fumigated with CP. These findings would be useful for establishing guidelines for biochar use in soil fumigation.
